# 

**Published:** 1893-03

**Authors:** 


					CONTENTS.
Acute Inflammation around the
upper Cervical Vertebrae.
By
John Kent Spender, M.D. Lond.
Pleural Effusions: their Diagnosis
and Treatment.
By Alfred
sheen, M.D. St. And.
14
A Case of Extra-Uterine Gestation.
By A. E. Aust Lawrence, M.D.
Aberd., and Charles A. Morton,
F.R.C.S. Eng
29
Progress of the Medical Sciences:
Medicine.
By J. Michell Clarke,
Surgery.
By W. M. Barclay
38
Laryngology and Rhinology.
By
B. J. Baron, M.B.
a
Reviews of Books
46
Notes on Preparations for the Sick
60
Society Meetings ...
G2
The Library of the Bristol Medico-
Chirurgical Society
64
Scraps
71

				

